# Effect of Aortic Valve Replacement on the Carotid Artery Distensibility in Patients with Aortic Valve Diseases

**DOI:** 10.1186/1749-8090-10-S1-A247

**Published:** 2015-12-16

**Authors:** Yukio Umeda, Yasunari Yokota, Yoshio Mori, Yoko Kawamura, Hiroshi Takiya

**Affiliations:** 1 Department of Cardiovascular Surgery, Gifu Prefectural General Medical Center, Gifu city, Gifu, 500-8717, Japan; 2 Faculty of Engineering, Gifu University, Gifu city, Gifu, 501-1193, Japan; 3 Research and Development Center for Human Medical Engineering, Gifu University, Gifu city, Gifu, 501-1193, Japan

## Background/Introduction

Impaired carotid artery distensibility in patients with aortic stenosis (AS) was reported previously. However, there is no physiological assessment of the carotid artery distensibility in patients with aortic valve diseases underwent aortic valve replacement (AVR).

## Aims/Objectives

In this study, we clarify the effect of AVR on the static and dynamic distensibility of the carotid artery in patients with aortic valve diseases.

## Method

Twenty patients with AS and 9 with aortic regurgitation (AR) underwent AVR were recruited. As static distensibility parameters of the carotid artery, such as β stiffness index, pressure-strain elastic modulus (Ep), and arterial compliance (AC), were obtained by a real time echo-tracking system at pre-AVR and 1 week after AVR. As a dynamic distensibility parameter of the carotid artery, maximum rate of rise of carotid diameter (dD/dt) was obtained.

## Results

Effective orifice area indices (EOAIs) were 1.13 ± 0.18 in AS group and 1.23 ± 0.12 in AR group, respectively. In AS group, post-AVR peak aortic velocities (AoV) was significantly reduced compared with pre-AVR peak AoV. On the other hand, post-AVR peak AoV was significantly increased compared with pre-AVR peak AoV in AR group.

There were no significant changes in the static distensibility parameters, such as β stiffness index, Ep, and AC, following AVR in each group. With regard to the dynamic distensibility parameters of the carotid artery in AS group, post-AVR dD/dt was higher than pre-AVR dD/dt (4.63 ± 1.80 mm/s vs. 3.30 ± 1.15 mm/s). In AR group, post-AVR dD/dt was lower than pre-AVR dD/dt (4.37 ± 0.95 mm/s vs. 9.33 ± 4.35 mm/s). In the evaluation of relationship between these dynamic distensibility parameters and the cardiac parameters for all AVR patients, there was significant correlation between the ratio of post- to pre-AVR peak AoV and the ratio of dD/dt (R = -0.753).

## Discussion/Conclusion

We found rapid and drastic alternation in dynamic distensibility of the carotid artery after AVR. It was affected by the alternation of peak AoV related to AVR.

